# Single-cell profiling reveals that SAA1+ epithelial cells promote distant metastasis of esophageal squamous cell carcinoma

**DOI:** 10.3389/fonc.2022.1099271

**Published:** 2022-12-20

**Authors:** Zhao Shu, Junfeng Guo, Qian Xue, Qi Tang, Bingqiang Zhang

**Affiliations:** ^1^ Department of Gastroenterology, The First Affiliated Hospital of Chongqing Medical University, Chongqing, China; ^2^ Department of Orthopaedics/Sports Medicine Center, State Key Laboratory of Trauma, Burn and Combined Injury, Southwest Hospital, Third Military Medical University, Chongqing, China

**Keywords:** esophageal squamous cell carcinoma, metastasis, scRNA-seq, SAA1, prognosis

## Abstract

**Introduction:**

Esophageal squamous cell carcinoma (ESCC) is one of the most common cancers globally, with significant cell heterogeneity and poor prognosis. Distant metastasis in ESCC is one of the key factors that affects the prognosis of patients.

**Methods and results:**

Starting with the analysis of ESCC single-cell sequencing data, we constructed a single-cell atlas of ESCC in detail and clarified the cell heterogeneity within tumor tissues. Through analysis of epithelial-mesenchymal transition (EMT) levels, gene expression, and pathway activation, we revealed the existence of a novel subpopulation of SAA1+ malignant cells in ESCC that are highly aggressive and closely associated with distant metastasis of ESCC. In vitro wound healing and transwell assays confirmed a strong invasion capacity of ESCC tumor cells with high expression of SAA1. Then, we constructed an effective and reliable prediction model based on the gene expression pattern of SAA1+ malignant cell subpopulations and confirmed that patients in the high-risk group had significantly worse prognosis than those in the low-risk group in the training cohort, internal verification cohort and external verification cohort.

**Discussion:**

This manuscript contributes to exploration of the heterogeneity of ESCC tumor tissues and the search for new ESCC subpopulations with special biological functions. These results contribute to our understanding of the underlying mechanisms of distant metastasis of ESCC and thus provide a theoretical basis for improved therapies.

## Introduction

Esophageal squamous cell carcinoma (ESCC) is one of the most aggressive squamous cell carcinomas and is particularly common in Asia (1). According to the latest Global Cancer Statistics 2020, esophageal cancer has the 7th highest incidence rate among all malignancies, with approximately 604,000 new cases and 544,000 deaths worldwide each year (2). ESCC is the most prevalent histological type of esophageal cancer, accounting for more than 90% of esophageal cancer cases (3). Despite the use of surgery, radiotherapy and chemotherapy, the prognosis for patients with ESCC remains poor, with a 5-year survival rate of only approximately 25% (4). In recent years, molecular-targeting therapy (including cetuximab and bevacizumab) and immunotherapy (including pembrolizumab and nivolumab) have been shown to effectively improve the survival and prognosis of patients with advanced esophageal cancer. However, the high costs and increased incidence of adverse reactions are arousing widespread concern (5).

Tumor metastasis is the leading cause of treatment failure in patients with ESCC (6). The poor outcome in esophageal cancer is largely due to cancer metastasis, with the 5-year survival rate declining from 43% for patients with localized disease to 23% and 5% for those with regional and distant metastasis, respectively (7). Epithelial-mesenchymal transition (EMT) is a reversible process in which epithelial cells acquire mesenchymal properties by changing their morphology, cellular structure, adhesion, and migration capacity. EMT initiates the metastatic properties of cancer cells by enhancing mobility, invasion, and resistance to apoptotic stimuli (8).

SAA1 is a member of the serum amyloid A family of apolipoproteins, which play an important role in chronic inflammation, cancer and other diseases (9). Xiao et al. found that serum SAA1 is a potential biomarker for eosinophilic granulomatosis with polyangiitis (10). Ren et al. discovered that SAA1+ epithelial cells were identified as a featured subpopulation of endometrial tumorigenesis (11). However, the role of SAA1 in the occurrence and development of ESCC has not been elucidated.

In this study, we constructed a single-cell atlas of ESCC tissue by an in-depth analysis of ESCC single-cell sequencing data. By comparing the EMT status, functional gene expression, and key pathway activation of different tumor cell subpopulations, we identified the crucial cell subset mediating ESCC metastasis and verified this finding in another independent sample dataset. These findings contribute to exploration of the heterogeneity of ESCC tumor tissues and the search for new ESCC subpopulations with special biological functions and provide a theoretical basis for further research on the molecular mechanisms related to ESCC progression and metastasis.

## Materials and methods

### ScRNA-seq data download and preprocessing

ESCC single-cell sequencing data were downloaded from the GEO database (GSE160269 and GSE188900). The original files were read by the CreateSeuratObject function in R and constructed into a Seurat object. We screened 1500 top variable features for further analysis, setting the selection method as “vst” (fitting a straight line to the relationship between log(variance) and log(mean) using local polynomial regression). We used the FindIntegrationAnchors function to find a set of anchors between different batches of data and then perform the dataset integration.

### Data dimensionality reduction and clustering

A combination of linear and nonlinear methods was employed to reduce the dimensionality of the data. First, principal component analysis (PCA) was conducted for linear dimensionality reduction, setting “weight.by.var” to TRUE to weight cell embeddings by the variance of each PC. Then, unified manifold approximation and projection (UMAP) was carried out for nonlinear dimension reduction, and the calculation method “uwot” was selected, and the parameter “min.dist” was set at 0.3. The k.param nearest neighbors were computed to construct the nearest-neighbor graph. The shared nearest neighbor (SNN) algorithm was used to identify clusters of cells.

### Assessment of EMT levels in epithelial cells

The genes related to EMT were obtained from the MSigDB database (GO:0001837). EMT scores were calculated for individual epithelial cells using the AddModuleScore function, with 100 control features selected from the same bin per analyzed feature. Single epithelial cells were colored on the UMAP dimensional reduction plot according to the EMT scores. Boxplots were drawn to compare the differences in EMT levels among different cell clusters.

### GSEA enrichment

GSEA software (v4.1.0) was downloaded from the official website (http://www.gsea-msigdb.org/gsea/index.jsp) and used for enrichment analysis. The gene set data were obtained from the MSigDB database and constructed into the GMT format file according to the instructions. The expression patterns of each subpopulation were calculated and disposed into input files in the RNK format. In the process of enrichment analysis, the number of permutations was set to 1000, and the permutation type was set to “gene set”.

### Cell culture

The human ESCC cell line KYSE-30 was generously provided by the American Type Culture Collection (Manassas, VA, USA). The cells were cultured in Dulbecco’s modified Eagle’s medium/nutrient mixture F-12 (DMEM/F-12; Gibco, CA, USA) supplemented with 10% fetal bovine serum (FBS; HyClone, UT, USA) and 1% penicillin−streptomycin (Sigma Aldrich, MO, USA) at 37°C with 5% CO2.

### Plasmid DNA transfection

The construction and amplification of the SAA1 plasmid were carried out by Genechem Co., Ltd. (Shanghai, China). The plasmid DNA vector was GV141 and the component sequence was CMV-MCS-3FLAG-SV40-Neomycin. Transfection was performed using INVI DNA Transfection Reagent. Specifically, 4 µg DNA was added to 50 µl of Opti-MEM and mixed gently. The transfection reagent (7 μL) was diluted in 50 µL of Opti-MEM. Then, the prepared DNA was combined with the prepared transfection reagent and incubated for 20 minutes at room temperature to allow the DNA-transfection reagent complexes to form. Finally, the complexes were added to each well.

### Real‐time quantitative PCR

Total RNA was extracted from cells using RNeasy Kits, according to the protocol provided by the manufacturer (QIAGEN, Hilden, Germany). The superscript III first‐strand synthesis kit (TaKaRa) was used to synthesize complementary DNA (cDNA) from total RNA. Then, RT-qPCR was performed on the ABI Prism 7900 Sequence Detection System (PE Applied Biosystems, Foster City, CA, USA) using a SYBR Green RT-PCR kit (TaKaRa). Expression levels were normalized to expression of the housekeeping gene glyceraldehyde-3-phosphate dehydrogenase (GAPDH).

### Wound healing

KYSE-30 cells were digested using 0.05% Trypsin‐EDTA (Sigma Aldrich, MO, USA), resuspended in the wash buffer at 4 * 10^5^ cell/ml, and then seeded into 24-well plates (2 * 10^5^ cell/well). When the cells reached approximately 90% confluence (about 24 hours after the seeding), the plate was vertically scratched with a 50 µL sterile pipette tip. Floating cells were removed by washing with PBS (1X) three times. The scratch was examined under an inverted microscope (IX73P2F, Olympus, Japan), and photographs were taken at 0 and 24 hours.

### Transwell assay

The transfected cells were digested using 0.05% Trypsin‐EDTA (Sigma Aldrich, MO, USA). Briefly, culture medium was removed, and cells were washed once with PBS. Remove PBS, add 200 µL of 0.05% trypsin‐EDTA, and incubate for 3 min at room temperature. When the fibroblasts showed cell contraction and increased cell space under the microscope, trypsin was removed and medium containing 10% FBS was added to stop the digestion. The cells were suspended by blowing and beating with a pipette and then collected in centrifugal tubes. Cells were counted and resuspended in serum-free medium (10 * 10^4^ cell/mL), and then 200 µL of cell suspension was added to each well (2 * 10^4^ cell). After 24 hours of incubation, the cells were fixed with a 4% paraformaldehyde solution and stained with 0.1% crystal violet.

### Grouping of ESCC patients

RNA-seq and clinical data from 176 ESCC patients were downloaded from GSE53625. These patients were randomly divided into two cohorts: a training cohort (n = 88) and an internal verification cohort (n = 88). In addition, ESCC sequencing data from the database TCGA were downloaded, which was used as an external verification cohort (n = 155). To ensure the accuracy of the results, we excluded patients who had been followed for less than 30 days.

### Risk score calculation

The regression model calculates a risk score for each patient based on the following formula:


$$Risk\;score=\;\sum_{i=1}^{n}(e_{i}*\;c_{i})$$

where N is the number of genes in the model; e_i_ is the gene expression; c_i_ is the gene coefficient in the regression model.

### Statistical analysis

Bilateral tests were performed for statistical tests. The mean ± standard deviation (SD) was used to present the quantitative data. A P value less than 0.05 was considered statistically significant, the statistical difference between groups is indicated on graphs with stars: the stars (from 1-4 stars) respectively represent p-values less than 0.05, 0.01, 0.001 and 0.0001. Some R packages were used in this study, including “limma”, “Seurat”, “dplyr”, “magrittr”, “infercnv”, “survival” and “survminer”.

## Results

### Cell type identification in the ESCC microenvironment

**Figure 1:**
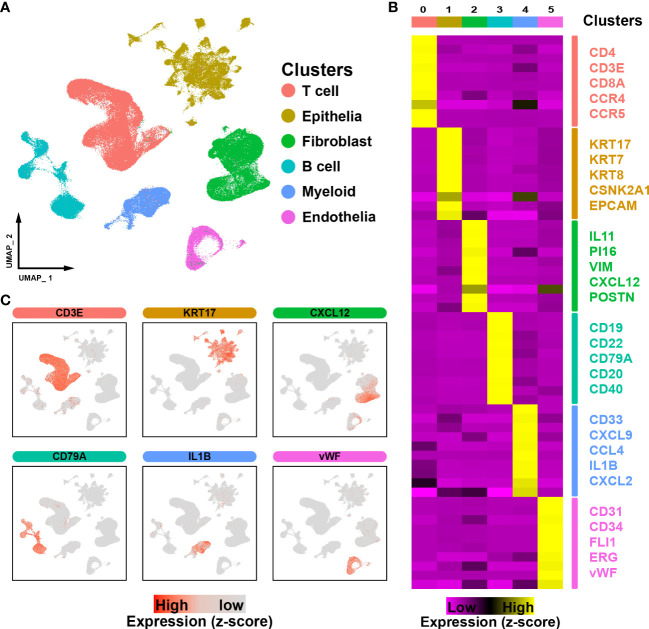
ScRNA-seq profiling of ESCC microenvironments. **(A)**. The UMAP plot visualizes the cell types in the ESCC microenvironment, with each cell type represented by a different color. **(B)**. Heatmap showing the expression of marker genes in the different cell types. **(C)**. UMAP plot showing the expression levels of marker genes for different cell types.

To perform an in-depth analysis of the cell composition within the tumor microenvironment in ESCC, a total of 206,701 cells with scRNA-seq data were examined in this study. Through unbiased clustering, we divided cells into 6 main clusters and used the UMAP algorithm to reduce dimensionality (**Figure 1A**). Cluster-specific markers were extracted and used for cell type identification: T cells (CD4, CD3E, CD8A, CCR4 and CCR5, 69324 cells); epithelial cells (KRT17, KRT7, KRT8, CSNK2A1 and EPCAM,43498 cells); fibroblasts (IL11, PI16, VIM, CXCL12 and POSTN, 41448 cells); B cells (CD19, CD22, CD79A, CD20 and CD40, 22524 cells); myeloid cells (CD33, CXCL9, CCL4, IL1B and CXCL2, 17346 cells) and endothelial cells (PECAM1, CD34, FLI1, ERG and vWF, 12561 cells) (**Figures 1B, C**).

### Heterogeneous analysis of the metastatic potential of malignant epithelial cells

**Figure 2:**
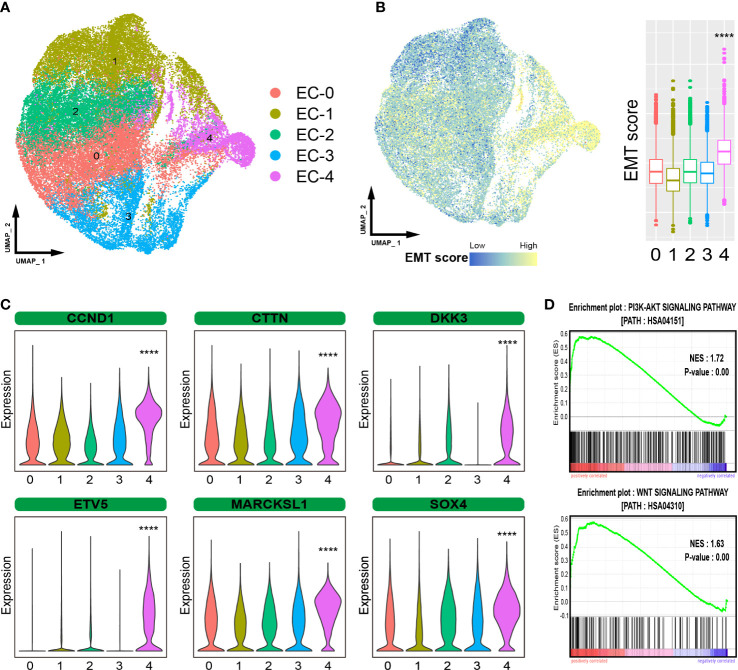
Heterogeneous analysis of malignant epithelial cells. **(A)**. UMAP plot showing the subpopulations of epithelial cells. **(B)**. EMT levels of epithelial cells. **(C)**. Violin plot showing the expression of certain metastasis-related genes. **(D)**. GSEA showing that the pathways activated in EC-4 cells. The symbol **** represent p-values less than 0.0001.

ESCC is characterized by the uncontrolled proliferation of epithelial cells. We further investigated epithelial cells to gain a better understanding of the cellular heterogeneity. A total of 43498 epithelial cells were subdivided into five subpopulations (EC-0 to EC-4, **Figure 2A**). Moreover, EMT is a crucial biological process in which epithelial-driven malignant tumor cells acquire the ability to migrate and invade (12). We evaluated the EMT levels in epithelial cells and found that cells in EC-4 showed the highest level of EMT, suggesting that these cells are tending to metastasize (**Figure 2B**). We detected the gene expression patterns of EC-4 cells and found that certain genes that have been proven to promote ESCC metastasis were significantly enhanced in the EC-4 group (CCND1, CTTN, DKK3, ETV5, MARCKSL1, SOX4, **Figure 2C**). In addition, the PI3K-AKT and WNT pathways were found to play an important role in promoting metastasis (13, 14). Gene Set Enrichment Analysis (GSEA) showed that these two pathways were significantly activated in EC-4 cells (**Figure 2D**). Collectively, these results pointed to EC-4 cells as a central driver of metastasis in ESCC, with stronger metastatic potential than other cell subpopulations.

### SAA1 is the primary target for highly invasive epithelial cells

**Figure 3:**
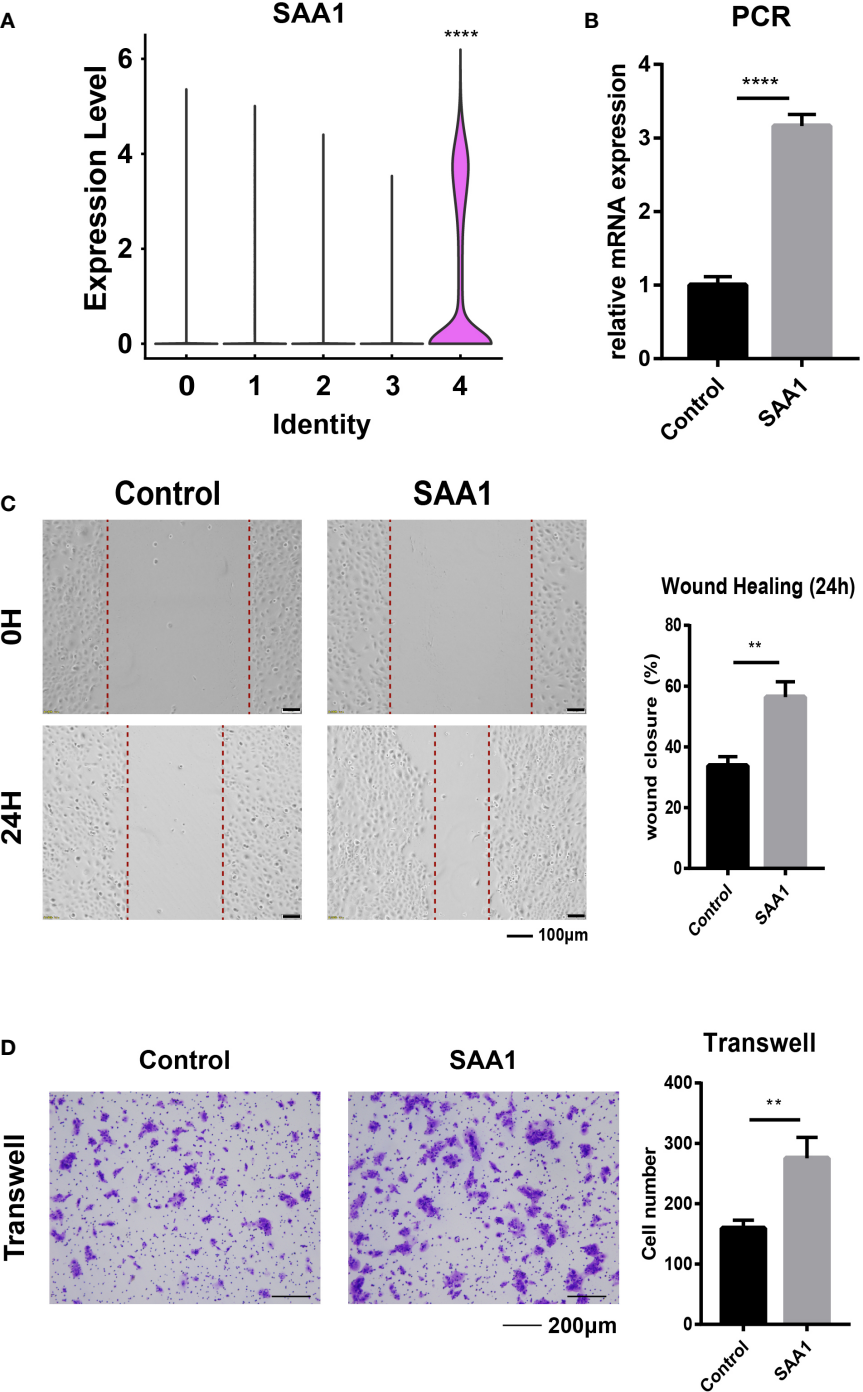
SAA1 is the primary target for EC-4. **(A)**. Violin plot showing the expression of SAA1 in different epithelial subpopulations. **(B)**. PCR analysis confirmed the overexpression of SAA1. **(C)**. Wound healing assays were performed to evaluate the migratory capacity after overexpression of SAA1. **(D)**. Transwell assays were performed to assess the invasion capacity after overexpression of SAA1. The symbols ** and **** represent p-values less than 0.01 and 0.0001.

To clearly define the highly invasive cell subpopulations in ESCC, we compared the gene positive rate of EC-4 cells to that of other cell subpopulations and found that SAA1 exhibited the most significant difference (**Figure S1A**). So, we defined the EC-4 group as SAA1+ epithelial cell subpopulations (**Figure 3A**). Survival analysis showed that ESCC patients with low SAA1 expression had a better prognosis than those with high SAA1 expression (**Figure S1B**). We are wondering whether SAA1 is the key gene that induces EC-4 cells to acquire the ability to be highly invasive. KYSE-30 expresses relatively low levels of SAA1(**Figure S1C**). The PCR results showed that we successfully increased the expression of SAA1 in the epithelial cell line KYSE-30 through plasmid transfection (**Figure 3B**). Wound healing assays confirmed that KYSE-30 cells with high SAA1 expression had stronger migration ability (**Figure 3C**). This stronger migration ability could be attenuated after SAA1 interference (**Figure S1D**). Similarly, transwell assays showed a significant increase in the number of highly invasive cells after overexpression of SAA1 (**Figure 3D**). These findings suggest that SAA1 plays a pivotal role in mediating the invasiveness of EC-4 cells.

### Independent verification of SAA1+ epithelial cell subpopulations

**Figure 4:**
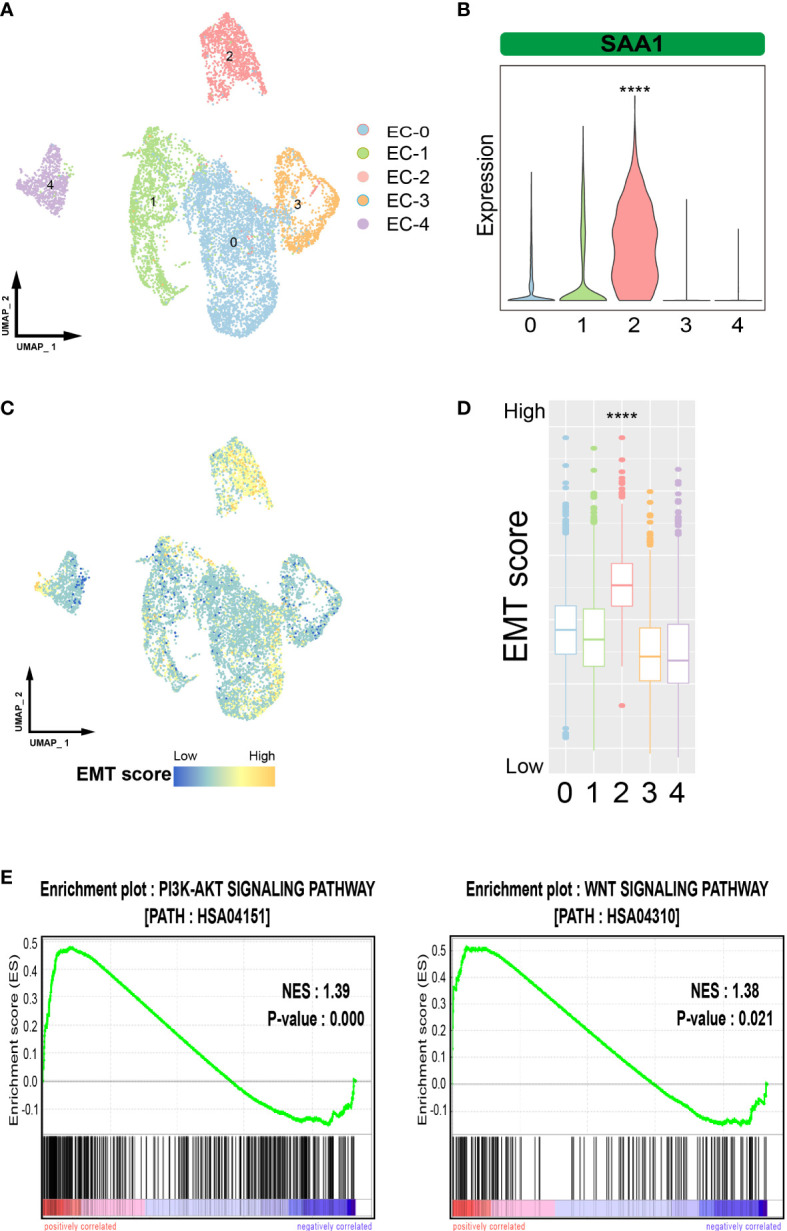
Verification of SAA1+ epithelial cell subpopulations. **(A)**. UMAP plot showing the subpopulations of epithelial cells in the verification data. **(B)**. Violin plot showing the expression of SAA1 in different epithelial subpopulations in the verification data. **(C)**. UMAP plot showing EMT levels of epithelial cells in the verification data. **(D)**. Box plot showing the EMT levels of epithelial cells in the verification data. **(E)**. GSEA showing that the pathways activated in SAA1+ cell subpopulation. The symbol **** represent p-values less than 0.0001.

To verify the existence of highly aggressive SAA1+ epithelial cell subpopulations, we imported another set of ESCC single-cell sequencing data. The 9181 tumor cells were divided into 5 cell subpopulations (**Figure 4A**). We examined the expression of SAA1 and found that it was predominantly expressed in cell subpopulation 2, suggesting that cell subpopulation 2 was the SAA1+ epithelial cells (**Figure 4B**). The other subpopulations also have similar corresponding relationships. Subpopulation 0 highly expressed the markers of EC-0 (ELF3, PHLDA2 and LYPD3); subpopulation 1 cluster cells overexpressed genes related to EC-3, such as S100A9 and B2M; subpopulation 3 cluster may predominantly correspond to EC-1 (STMN1, TUBB, HMGB1) and subpopulation 4 corresponded to EC-2 (DSC3, LAMTOR4, RPS11) (**Figure S2**). Upon further evaluation of the metastasis-related characteristics of this cell subpopulation, we confirmed that EMT scores were significantly higher in cell subpopulation 2 than in the others, as expected (**Figures 4C, D**). Meanwhile, GSEA showed that the PI3K-AKT and WNT pathways were also significantly activated in cell subpopulation 2 (**Figure 4E**). In summary, these results show that a highly aggressive SAA1+ epithelial cell subpopulation does exist in ESCC.

### Establishment of the risk regression model based on EC-4 cells

**Figure 5:**
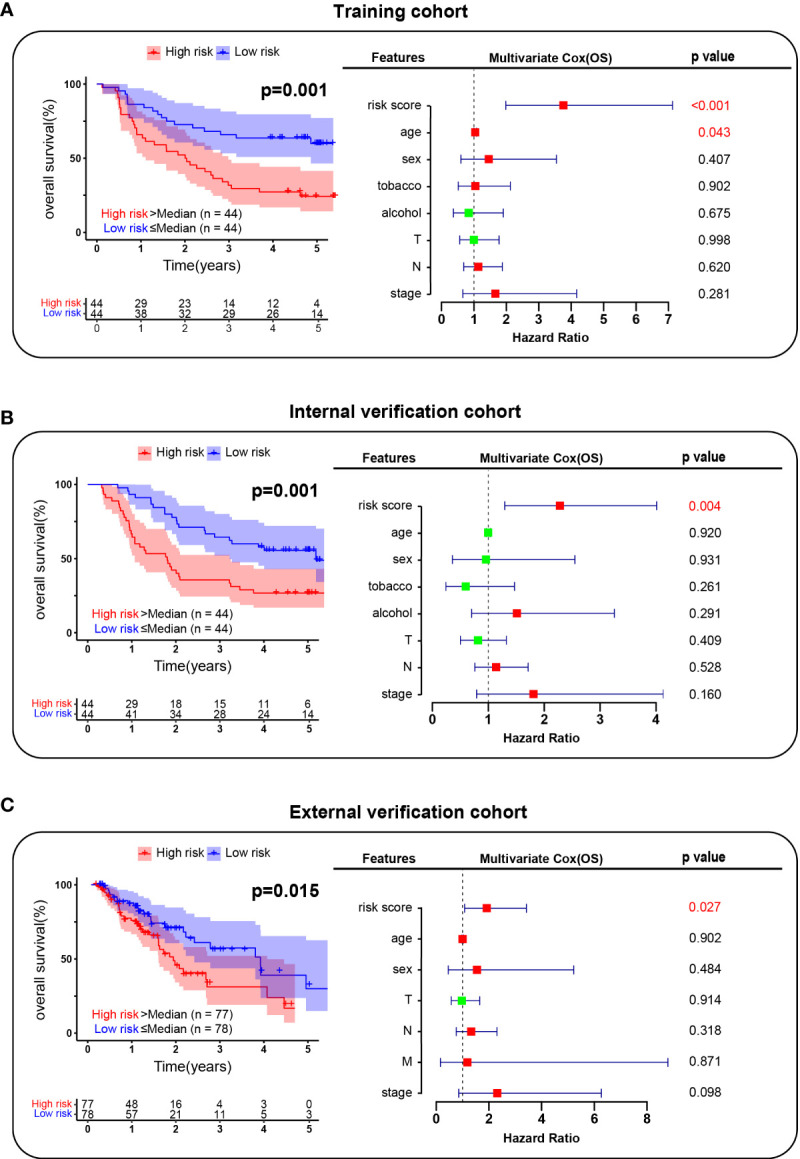
Risk regression model establishment. **(A)**. Survival analysis of patients in the training cohort. **(B)**. Survival analysis of patients in the internal verification cohort. **(C)**. Survival analysis of patients in the external verification cohort. The red box indicated the risk factor and the green indicated the protective factor.

Furthermore, we hope to use the characteristics of EC-4 cells to predict the prognosis of ESCC patients. We analyzed the gene expression patterns of EC-4 subpopulations and collected a total of 602 EC-4-specific genes (including 322 upregulated genes and 280 downregulated genes). The results of the univariate Cox analysis identified the genes which was significantly associated with the prognosis of patients in the training cohort. The 8 genes EC-4-related prognostic model was established by lasso regression analysis, including CTTN, SSPN, GRB7, FOXP1, SNX1, ALDH7A1, CXCL14 and PODXL2. We applied the model to predict the survival of patients in the training cohort. The results showed that the prognosis of patients in the low-risk group was significantly better than that in the high-risk group (**Figure 5A**). The risk score yielded a c-index of 0.768 (95% CI, 0.740-0.796) in the training cohort. The multivariate Cox results showed that the risk score was an independent predictor of prognosis in ESCC patients, and its predictive ability was superior to that of traditional clinical assessment indicators (age, sex, smoking, alcohol and TNM stage). Further extending the application range, our prognosis model exhibited great performance in both the internal verification cohort and the external verification cohort (**Figures 5B, C**). The risk score had a c-index of 0.730 (95% CI, 0.700- 0.761) in the internal verification cohort and 0.707 (95% CI, 0.673- 0.741) in the internal verification cohort. In general, these results confirmed that gene expression patterns of EC-4 subpopulations could accurately reflect the developmental characteristics of ESCC and that making a risk regression model using the EC-4 expression profiles could effectively predict the prognosis of ESCC patients.

## Discussion

ESCC is one of the most invasive tumors with a high incidence rate (15). Despite surgery, chemoradiotherapy and molecular-targeting therapy, most patients with ESCC develop metastasis, which leads to treatment failure (16). Elucidating the molecular mechanism of ESCC metastasis is crucial to improve the treatment method, prevent tumor metastasis and ameliorate the prognosis. In this study, we analyzed the cellular composition of the ESCC tumor microenvironment through in-depth mining of ESCC single-cell sequencing data. We found that in addition to malignant epithelial cells, tumor tissues contain a large number of immune cells (T cells, B cells, and myeloid cells), which suggests that tumor immunity plays an important role in the occurrence and development of ESCC. Th1 cells have been shown in previous studies to upregulate interferon-γ response signaling and antigen presentation pathways and downregulate lipid metabolism and MAPK pathways of ESCC cells, thereby improving the neoadjuvant chemoradiotherapy response of esophageal squamous cell carcinoma (17). Wang et al. found that the characterization of the intratumor B-cell immunoglobulin repertoire could help to predict the prognosis of ESCC (18).

As the main cell type of tumor metastasis, malignant epithelial cells are the focus of this study. Abnormal activation of EMT is critical for cancer cells during tumor progression and metastasis (19). In the malignant epithelial cells of ESCC, we identified a novel subpopulation (EC-4) that exhibited an extremely high tendency to undergo EMT. Many key genes closely associated with ESCC metastasis show widely dysregulated expression patterns in EC-4, such as CTTN. CTTN is an oncogene that promotes the metastasis of ESCC. It binds to and activates the actin-related protein complex (Arp2/3), thereby modulating the actin branching network to form dynamic cortical actin-related structures (20).

SAA1 was identified as a marker gene of EC-4 cells. Previous studies have confirmed its close association with chronic inflammation, SAA1+ cell subpopulations may be extensively related to the inflammatory response of ESCC, which is worth further study (21). Moreover, SAA1 has been widely studied in a variety of tumors. In oral cancer, SAA1 promotes tumor metastasis by inducing EMT (22). Cancer-associated adipocytes affect the progression of pancreatic cancer by regulating the expression of SAA1 (23). Additionally, high expression of SAA1 can be used as an effective predictor of advanced renal cell carcinoma (24). We confirmed *in vitro* that ESCC cells with high SAA1 expression were more invasive and migratory by wound healing and transwell assays, and these results supported our hypothesis that SAA1+ ESCC cell subpopulations have unique biological functions.

Many studies have predicted ESCC patient prognosis in a variety of ways. Yu et al. revealed diagnostic biomarkers and risk factors for esophageal squamous cell carcinoma by plasma metabolomics. However, it is unclear whether the findings are applicable to other regions and populations, as the study was conducted in a single center. Zhu et al. constructed a prognostic model for ESCC patients based on lncRNAs, but the model contained too many genes, which was not conducive to wide clinical application (25). With the rapid development of single-cell sequencing technology, the heterogeneity of tumor cells has been widely studied. Our study combined the advantages of both single-cell sequencing and transcriptome sequencing and successfully developed a metastasis-related prognostic model based on the expression pattern characteristics of the SAA1+ highly invasive subpopulation of ESCC. This model not only has good performance in the training group but also showed excellent prediction ability in the independent internal and external verification cohorts.

In conclusion, we mapped the tumor microenvironment by mining ESCC single-cell sequencing data. Through the assessment of EMT levels in epithelial cells and the comprehensive analysis of key genes and pathways, we found a group of SAA1+ malignant epithelial cells in esophageal cancer that are highly invasive and play an important role in the distant metastasis of ESCC. Based on the results, we constructed an ESCC metastasis-related prognostic model that could accurately assess patient prognosis. These findings contribute to our understanding of the underlying mechanism of ESCC metastasis and further improve the treatment and prognosis of patients with ESCC.

## Data availability statement

The original contributions presented in the study are included in the article/**Supplementary Material.** Further inquiries can be directed to the corresponding author.

## Author contributions

Conceptualization, BZ. Methodology, BZ. Software, ZS. Validation, ZS and JG. Formal analysis, ZS and JG. Investigation, QX. Resources, QT. Data curation, JG. Writing-original draft preparation, ZS and JG. Writing-review and editing, BZ. Visualization, JG and ZS. Supervision, QT. Project administration, BZ. Funding acquisition, BZ. All authors have read and agreed to the published version of the manuscript.
