# Supplementary material for: Single-cell profiling reveals that SAA1+ epithelial cells promote distant metastasis of esophageal squamous cell carcinoma
Source: Front Oncol. 2022 Dec 20;12:1099271. doi: 10.3389/fonc.2022.1099271 (PMC9807783; doi:10.3389/fonc.2022.1099271)
Supplement: Supplementary file 1 [file DataSheet_1.docx]

Supplementary Material

# Supplementary Table 1. The gene expression and the gene coefficient in the regression model.

| Gene | Coef |
| --- | --- |
| CTTN | 0.342 |
| SSPN | 0.134 |
| GRB7 | -0.199 |
| FOXP1 | 0.246 |
| SNX1 | 0.195 |
| ALDH7A1 | 0.032 |
| CXCL14 | -0.066 |
| PODXL2 | -0.171 |


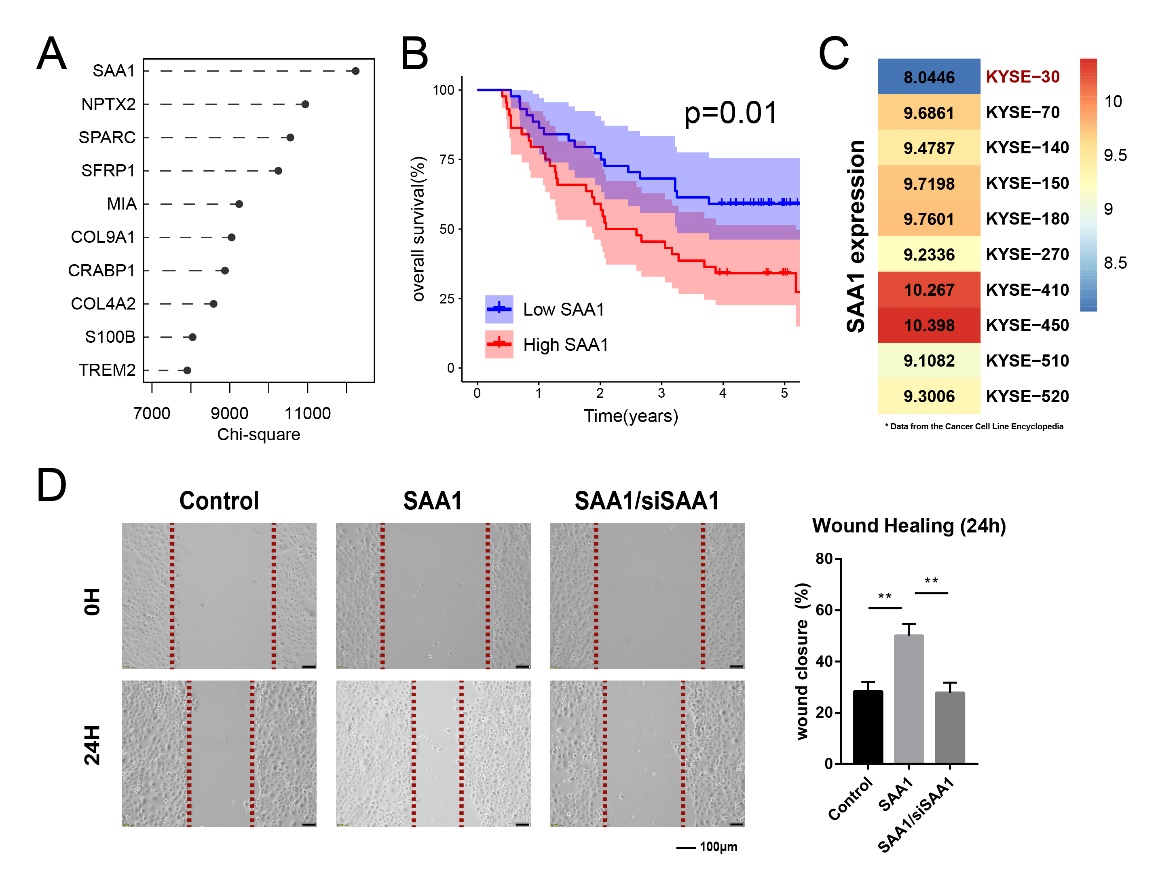


**Supplementary Figure 1. SAA1 is the primary target for highly invasive epithelial cells. A.** SAA1 exhibited the most significant difference. **B.** Survival analysis of SAA1 in ESCC. **C.** SAA1 expression levels in different ESCC cell lines. **D.** The migration ability was attenuated after interference with SAA1.


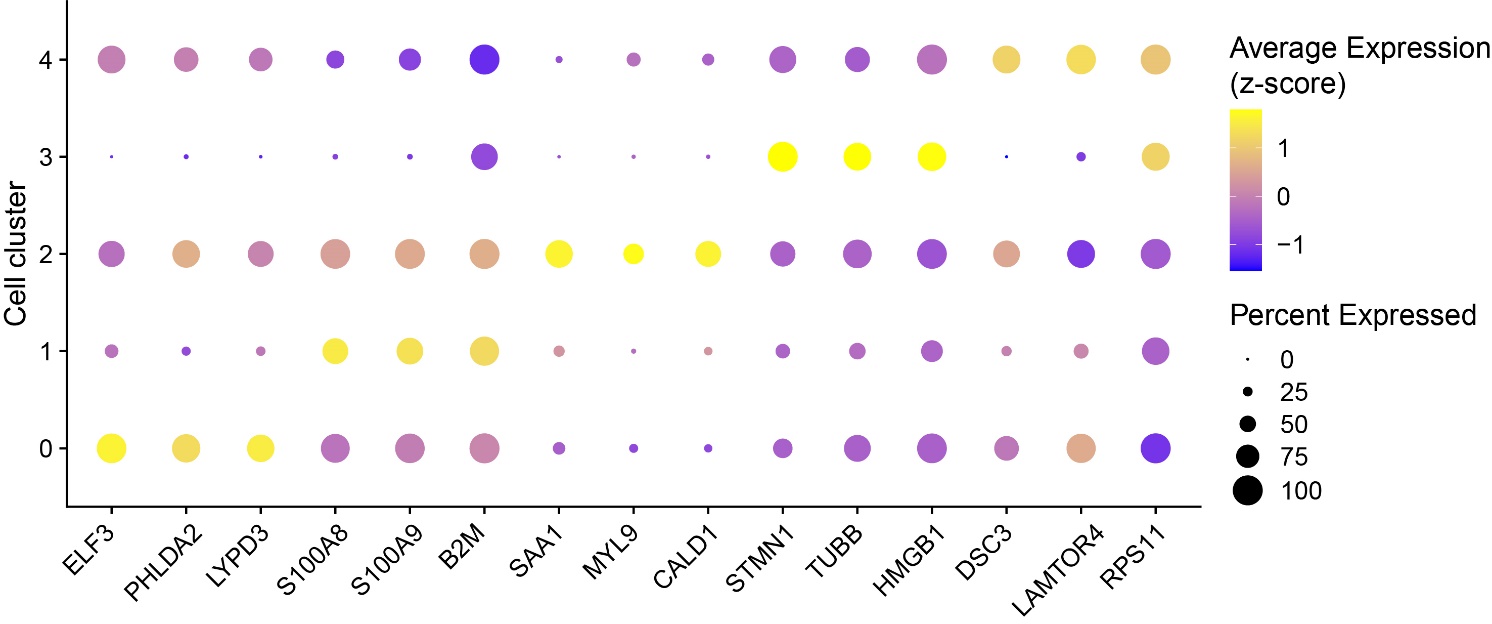


**Supplementary Figure 2. The marker genes of epithelial cell subpopulations in independent verification data.**
